# Psychotherapeutic Interventions in Irritable Bowel Syndrome

**DOI:** 10.3389/fpsyt.2020.00286

**Published:** 2020-04-30

**Authors:** Larissa Hetterich, Andreas Stengel

**Affiliations:** ^1^Department of Psychosomatic Medicine and Psychotherapy, University Hospital Tübingen, Tübingen, Germany; ^2^Department for Psychosomatic Medicine—Germany, Charité Center for Internal Medicine and Dermatology, Corporate Member of Freie Universität Berlin, Berlin Institute of Health, Charité - Universitätsmedizin Berlin, Humboldt-Universität zu Berlin, Berlin, Germany

**Keywords:** brain-gut axis, hypnotherapy, psychodynamic, psychoeducation, psychosomatic

## Abstract

Irritable bowel syndrome (IBS) is a frequent functional gastrointestinal disorder. The patients complain about various symptoms like change in bowel habits, constipation or diarrhea, abdominal pain, and meteorism leading to a great reduction in quality of life. The pathophysiology is complex and best explained using the biopsychosocial model encompassing biological, psychological as well as (psycho)social factors. In line with the multitude of underlying factors, the treatment is comprised of a multitude of components. Often, patients start with lifestyle changes and dietary advice followed by medical treatment. However, also psychotherapy is an important treatment option for patients with IBS and should not be restricted to those with psychiatric comorbidities. Several evidence-based psychotherapeutic treatment options exist such as psychoeducation, self-help, cognitive behavioral therapy, psychodynamic psychotherapy, hypnotherapy, mindfulness-based therapy, and relaxation therapy which will be discussed in the present review.

## Introduction

Irritable bowel syndrome (IBS) is a functional disorder of the large bowel (1). In the ICD-10 it is categorized within the functional disorders, in the ICD-11 it will be found in the section of bodily distress disorders. Patients with IBS can present with a wide array of symptoms such as abdominal distension, meteorism and flatulence, abdominal pain as well as a change in bowel habits such as constipation or diarrhea (2). The prevalence of IBS varies greatly with 1 to 45%—most likely due to different diagnostic criteria applied—with an average worldwide prevalence of 11.2% (3), well reflecting the prevalence in western countries with 10–20% (4).

IBS can be diagnosed worldwide using the Rome criteria (last revised in 2016 and termed Rome IV) when the patient complains of the main symptom being recurring abdominal pain that occurred during the last 3 months not less than once per week (2). Additionally, two of the following three criteria have to be fulfilled:

- The complaints are associated with defecation,
- The complaints are associated with change in frequency of defecation and
- The complaints are associated with change in consistency of stool (2).

According to the Rome IV criteria IBS can be classified into four different subgroups:

- IBS-D (diarrhea): >25% of the stool is fluid, without solid components, <25% are solid components,
- IBS-C (constipation): >25% of the stool are separate solid clots, <25% fluid, without solid components,
- IBS-M (mixed): >25% of the stool are fluid, without solid components and >25% are separate solid clots,
- IBS-U (unclassified): not clearly allocable (2).

These complaints are very often associated with a great reduction in quality of life. A study from 2014 showed that quality of life in patients with IBS depends on different parameters such as clinical variables (24%), fear of gastrointestinal symptoms (14%) or demographics (10%). Also psychiatric disorders may be a consequence of the disease (5). This underlines the need for a proper treatment offer (6).

In the present review we evaluated the state-of-knowledge on psychological treatment options for patients with IBS and as well discussed gaps in knowledge in order to foster further research. We employed the following databases: PubMed and ScienceDirect using these keywords: brain-gut axis, cognitive behavioral therapy, hypnotherapy, IBS, irritable bowel syndrome, mindfulness-based therapy, psychodynamic, psychoeducation, psychosomatic, relaxation therapy, and self-help. Only human studies were considered for the review, while animal studies were excluded. The search was conducted for articles from 1983 to 2019, the discussion highlights recent developments.

## Pathophysiology

The pathogenesis of IBS is complex and best explained using the biopsychosocial model encompassing biological, psychological, and (psycho)social factors (7) that can all contribute to the development and maintenance of the disease. In line with this model, genetic and environmental factors can affect the disorder as well as special personality traits (8). These traits can contribute to coping strategies which, when not sufficient anymore, might also facilitate development of the disease. Various psychosocial factors are important for IBS e.g., early life experiences, infections, trauma, stress, cultural background, and also the level of support the individual receives. Negative life experiences are considered an important risk factor for the development of IBS. People who experienced more (severe or frequent) negative life events show a higher prevalence of IBS and might have a more severe progress of the disorder (9).

The gut-brain-axis is a bidirectional communication system between the gut and the central nervous system (10). Afferent nerve pathways as well as humoral signals transmit information from the gastrointestinal tract to the central nervous system. The information gets processed in various brain areas and often feedbacks back to the gut. A dysregulated gut-brain-axis can lead to e.g., altered bowel motility, intestinal immune reaction, or intestinal permeability which may drive inflammatory responses that may contribute to visceral hypersensitivity (11). Also, the microbiome may likely play a role in the pathophysiology of IBS since the microbiome of patients with IBS seems to be less variable (12) and might be located in other parts of the gut as seen in small intestinal bacterial overgrowth (13). Due to the impact of the microbiome on gastrointestinal as well as central processes, the term microbiome-gut-brain axis was introduced. The importance of the gut-brain axis in the pathophysiology of IBS is also reflected in the Rome IV criteria where IBS has been allocated to diseases with discorded gut-brain interaction (14). The intimate interaction between gut and brain also explains the high overlap between IBS and psychiatric diseases which also has an impact on the selection of the treatment components. Most frequent psychiatric comorbidities are anxiety disorders (30–50%), depression (70%), but also—although less frequent—eating disorders (5).

## Psychotherapy

Besides lifestyle changes, dietary advice, and drug treatment, psychotherapy is an important column in the treatment of IBS. Not every patient has to undergo psychotherapy but especially in patients with insufficient social support, traumatic events in their history, or dysfunctional relations, psychotherapy should be considered early on (15). Moreover, patients with psychiatric comorbidities (5) or those that do not show significant improvement after treatment with other treatment options (e.g., drugs) (16) should be considered for psychotherapy. The National Institute of Health and Care Excellence (NICE) guidelines recommend a psychological therapy for patients who do not respond to pharmacological treatments after 12 months and develop refractory IBS. Psychotherapy options contain psychoeducation, self-help, cognitive behavioral therapy, psychodynamic psychotherapy, hypnotherapy, mindfulness-based-therapy, and relaxation therapy (17) which will be discussed in this review. The likelihood for a psychological intervention to be successful is greater in patients that are motivated and open for psychotherapy (18), although this should be the prerequisite for offering this therapy.

A meta-analysis showed the benefit of pooled psychological interventions in patients with IBS with a very low number needed to treat (NNT) of 2 (18). Similarly, a recent meta-analysis calculated a NNT of 4 (95% confidence interval, CI, 3.5–5.5) (19). While the NNT suggests a very prominent effect of psychotherapy, a systematic review reported that the psychotherapy-induced positive effects on symptoms did not last longer than the positive effects induced by other treatment options such as medication (20). A more recent meta-analysis included 41 trials with overall 2,290 individuals and compared psychological interventions with a mix of control conditions. It was shown that psychotherapeutic interventions decreased the symptoms immediately after the treatment, while 1–6 months (short-term) and 6–12 months (long-term) after start of the treatment the reduction remained significant compared to the control group(s) (21). Additionally, another meta-analysis analyzed the effect of psychotherapeutic interventions on mental health and the daily functioning of patients. All psychotherapeutic interventions showed a greater improvement of mental health and daily functioning of IBS patients compared to a mixed group of control conditions (22). The next paragraphs will provide an overview on the effects of different psychotherapeutic techniques in the treatment of IBS.

### Psychoeducation

It is important that the physician takes time to explain the medical condition. This contains the name of the disease, development, pathophysiology, prognosis, and various treatment options (23). If this information is provided according to the biopsychosocial model, it would be referred to as psychoeducation (7). Moreover, psychoeducation is key for a trustful doctor-patient-relationship which greatly impacts on the course of the disease and reduction of symptoms, respectively (24). Psychoeducation can also help to reduce/avoid unnecessary repetitive (and sometimes invasive) examinations and/or non-suited therapeutic interventions as this could increase the probability of an incorrect concept of the disorder and potentially harm the patient (25). Therefore, the world gastroenterology organization mentions in its global guidelines that educating the patients about IBS has a positive effect on the treatment outcome (26).

A study used psychoeducation with information about pathophysiology in combination with elements of cognitive behavioral therapy (CBT) and progressive muscle relaxation for 5 weeks. After this psychoeducation, patients reported a significant reduction of somatic complaints and depressive symptoms and an improvement in quality of life compared to the control group. These effects persisted after a follow-up of 3 months (27).

Another study used a group education based on the biopsychosocial model with aspects of CBT compared to a control group that used an IBS manual. The group-educated patients reported a significant increase of knowledge after 3 and 6 months compared to the group using the manual. There was a reduction of symptom-specific anxiety in the group education group, while no significant differences between groups were seen with regards to quality of life, anxiety symptoms, and depressive complaints. Nevertheless, there was an increase of quality of life and reduction of anxiety in the group-educated group compared to the beginning of the treatment (28) (**Table 1**).

**Table 1:** Randomized controlled studies investigating the effects of psychoeducation in patients with irritable bowel syndrome.

Study	Population	Variables	Intervention	Results
Ringström et al. (2010) Structured patient education is superior to written information in the management of patients with irritable bowel syndrome: a randomized controlled study. *Eur J Gastroenterol Hepatol*. (28)	143 (87% female)	Quality of life Anxiety Depressive symptoms	Group education *vs.* IBS-manual	Group education: Increase of knowledge after 3 and 6 months, reduction of symptom-specific anxiety. No difference in groups: quality of life, anxiety, depressive complaints.
Labus et al. (2013) Randomised clinical trial: symptoms of the irritable bowel syndrome are improved by a psychoeducation group intervention. *Aliment Pharmacol Ther*. (27)	69 (72% female)	Somatic symptoms Depressive symptoms Quality of life	5 weeks: psychoeducation + elements of CBT + progressive muscle relaxation *vs.* control group	After treatment: significant reduction of somatic complaints, depressive symptoms, and improvement of quality of life.

### Self-Help

Self-help can be supported by detailed consultation and information about IBS e.g., with manuals or guidebooks. The patients can learn about experiences, coping strategies, or treatment options. This encourages the patients' self-management (29).

A randomized, controlled trial studied the effect of different self-help methods on the frequency of doctor's appointments and on symptom severity. One group received a self-help-manual, the other additionally visited a self-help group once per week. These groups were compared to a control group with routine outpatient care. Compared to the control group, the group with the self-help-manual reduced doctor's appointments (60% less in 1 year) associated with a reduction in health costs (40% less). The intervention group showed a significant improvement of complaints compared to the start of the study (Cohen's d=0.51). However, there was no difference related to symptom severity and quality of life between groups (30). Another trial studied the effect of a self-help manual on health-associated quality of life. After 6 months of intervention there was a significant improvement in quality of life. This effect was also observed in patients with psychiatric comorbidities like depression, anxiety, or somatization disorders. The severity of the psychiatric comorbidity was reduced over time (31) (**Table 2**).

**Table 2:** Randomized controlled study investigating the effects of self-help in patients with irritable bowel syndrome.

Study	Population	Variables	Intervention	Results
Robinson et al. (2006) A randomised controlled trial of self-help interventions in patients with a primary care diagnosis of irritable bowel syndrome. *Gut*. (30)	420 (89% female)	Symptom severity Frequency of doctor's appointments	Self-help manual *vs.* self-help manual + 1x/week self-help group *vs.* control group (outpatient care)	No difference between self-help groups; reduction of doctor's appointments (60% less) and health costs (40%) compared to control group; improvement of symptoms compared to beginning.

A meta-analysis examined the effect of self-help and self-management methods. There was a medium effect size (d=0.72) for the reduction of symptom severity and a large effect size (d=0.84) for the improvement of quality of life (32). Online interventions were especially suited to reduce somatic complaints and improve quality of life (32). However, it is to note that several studies do not have adequate control groups, the study population is often very small and blinding often not possible.

### Cognitive Behavioral Therapy

Most of the psychological interventions in the treatment of IBS are based on CBT aiming at the reduction of irrational fears and the modulation of behavioral patterns. The NNT with CBT was 4 (95% CI 3–9) (32). However, although CBT shows good results, it is not always available and labor-intensive. Therefore, the application also depends on the patient's intention, the expertise of the professional and the resources available (26).

A meta-analysis from 2019 studied nine randomized controlled trials (RCTs) compared to control groups with 610 patients in total. In 145 of 349 (41.5%) patients undergoing CBT the symptoms did not improve, compared to 166 of 261 (63.6%) patients in the control groups (19) indicating a beneficial effect of CBT.

A trial showed a 50% reduction of gastrointestinal symptoms, anxiety, and depression in the CBT group compared to symptom scores assessed at baseline (33). Another study investigated a combination of progressive muscle relaxation, cognitive behavioral strategies, and problem-solving approaches in comparison to a standardized medical treatment with drugs and regular appointments with a gastroenterologist. The group with the extended psychological treatment showed a decrease of bowel symptoms and an increase of well-being, quality of life and control of disease after 3 and 6 months compared to the control group whose symptoms remained unchanged (34). This study showed that the combination of drug therapy and psychological interventions is superior to medical treatment alone.

A more recent RCT used an online program based on CBT. A total of 86 patients were included and randomized to the control (an online discussion forum) or treatment group. The main measures were IBS symptom severity, quality of life, anxiety, depression, and general functioning. Patients in the treatment group reported a 42% decrease in IBS symptoms in comparison to the control group that reported a 12% increase in IBS symptoms (35). The follow-up after 15 and 18 months also showed that the group with the psychological intervention benefited with regards to symptoms, quality of life and anxiety (d=0.78–1.11) (36). Similarly, an internet-based treatment with CBT exerted positive effect in adolescents with IBS. A controlled study showed after an intervention of 10 weeks a decrease of gastrointestinal symptoms (d=0.45, NNT 4) and an improvement in quality of life (d=0.40) in the group with online psychotherapy in comparison to the control group (37). The symptoms of anxiety and depression also decreased in the course of the intervention but there was no significant difference between the therapy group and the control group (37). The follow-up after 6 months showed that the effects remained constant. Fear-characteristics, quality of life and frequency of pain further decreased during the follow-up (37).

A large study in 436 IBS patients allocated to either standard CBT (10 weekly sessions, 60 min/session with information on brain-gut interaction, self-monitoring symptoms, muscle-relaxation), four sessions of primarily home-based CBT with minimal therapist contact (MC-CBT), or four sessions of IBS education (EDU). After 12 weeks, a higher proportion of patients with MC-CBT reported an improvement in gastrointestinal symptoms (61%) than patients with EDU (43%), while 55% patients in the CBT group showed an improvement (16). At 6 months after the end of treatment, a significant difference was observed between MC-CBT (58.4%) and EDU (44.8%) with regards to improvement of bowel symptoms. Both CBT methods (CBT and MC-CBT) showed significantly higher patient satisfaction than EDU (d for MC-CBT = 0.53). The results showed that MC-CBT is as efficacious as standard CBT (16). Therefore, CBT might be offered also on a telemedical basis with minimal therapist contact, probably in an (even more) cost effective manner. In line with this assumption, another study investigated telephone-delivered CBT (TCBT) and web-based CBT (WCBT) in comparison to treatment as usual (TAU). The study showed that both CBT methods led to an improvement in IBS severity and coping strategies compared to TAU (38). Both methods were cost-effective.

A recent trial from 2019 with 60 IBS patients with diarrhea-predominant IBS and 30 healthy controls studied the effect of CBT and exercise on the coping styles and cognitive bias of patients (**Table 3**). The patients were divided into two groups: experimental group (CBT + exercise) and control group (conventional drugs). After 6–24 weeks there was an improvement of neglect and pain behavior along with a difference in perfectionism, dependence and vulnerability (47). This study shows that CBT in combination with exercise can help to change the cognitive bias and coping styles of patients with diarrhea-predominant IBS. Lastly, a study using rectal barostat showed that although CBT did not alter visceral discomfort, urge, and pain during barostat testing, self-rated visceral sensitivity did improve after CBT (48).

**Table 3:** Randomized controlled studies investigating the effects of cognitive behavioral therapy in patients with irritable bowel syndrome.

Study	Population	Variables	Intervention	Results
Greene & Blanchard (1994) Cognitive therapy for irritable bowel syndrome. *J Consult Clin Psychol*. (39)	20 (75% female)	GI symptoms	2 weeks: 2x 1 h intervention/week, 6 weeks: 1x 1 h intervention/week *vs.* control group with symptom monitoring	Post treatment: 80% of CBT group and 10% of control group with significant improvement of GI symptoms.
Payne & Blanchard (1995) A controlled comparison of cognitive therapy and self-help support groups in the treatment of irritable bowel syndrome. *J Consult Clin Psychol*. (33)	22 (82% female)	Individual GI symptoms Composite index for GI symptoms	2 weeks: 2x 1 h intervention/week, 6 weeks: 1x1 h intervention/ week *vs.* symptom-monitoring waiting-list control	50% reduction of gastrointestinal symptoms, anxiety, and depression in the CBT-group compared to baseline symptom score.
Vollmer & Blanchard (1998) Controlled comparison of individual *versus* group cognitive therapy for irritable bowel syndrome. *Behav Ther*. (40)	34 (76% female)	Clinical symptoms	10 weeks: 1 h individual CBT session/week or 10 weeks: 90 min group CBT session/week or monitoring (control group)	Post treatment: improvement of clinical symptoms: 64% in group CBT, 55% in individual CBT, 10% in control group.
Heymann-Mönnikes et al. (2000) The combination of medical treatment plus multicomponent behavioral therapy is superior to medical treatment alone in the therapy of irritable bowel syndrome. *Am J Gastroenterol*. (34)	21 (87.5% female)	IBS symptoms Well-being Quality of life	10 weeks: 1x1 h session multicomponent behavioral therapy/ week + medication *vs.* control group: medication only	Improvement in the behavioral therapy group (well-being, quality of life, symptoms; no change in the control group).
Boyce et al. (2003) A randomized controlled trial of cognitive behavior therapy, relaxation training, and routine clinical care for the irritable bowel syndrome. *Am J Gastroenterol*. (41)	105 (81% female)	General health Pain Physical functioning Anxiety Depression	8 weeks: 1x 1 h CBT/week *vs.* 8 weeks: 1x 30 min relaxation therapy/week	Reduction in anxiety, depression, improvement of general health, pain and physical functioning, no difference between groups.
Drossman et al. (2003) Cognitive-behavioral therapy *versus* education and desipramine *versus* placebo for moderate to severe functional bowel disorders. *Gastroenterology*. (42)	169 (100% female)	Clinical, physiological, and psychosocial assessment	12 weeks: 1x 1 h CBT/week *vs.* control group (education)	CBT was more beneficial over Education for all parameters except for depressiveness.
Tkachuk et al. (2003) Randomized controlled trial of cognitive-behavioral group therapy for irritable bowel syndrome in a medical setting. *J Clin Psychol Med Settings*. (43)	28 (96% female)	Global symptoms	1 week: 2x 90 min group CBT intervention, 8 weeks: 1x 90 min group CBT intervention/week *vs.* home-based symptom monitoring	Better improvement in global symptoms, daily pain, psychological distress, and quality of life in CBT group.
Kennedy et al. (2003) Cognitive behaviour therapy in addition to antispasmodic treatment for irritable bowel syndrome in primary care: randomised controlled trial. *BMJ*. (29)	149 (n.s.)	Work and social adjustment scale Symptom severity	6 weeks: 1x 50 min CBT/week + mebeverine *vs.* control group (mebeverine only)	CBT showed better reduction of symptom severity, benefit on work, and social adjustment scale compared to control group; effects persisted after 6–12 months.
Lackner et al. (2008) Self-administered cognitive behavior therapy for moderate to severe irritable bowel syndrome: clinical efficacy, tolerability, feasibility. *Clin Gastroenterol Hepatol*. (44)	75 (87% female)	IBS symptom severity Quality of life Global symptoms	10 weeks: 1x 1 h CBT/week *vs.* 10 weeks: 1x 1 h CBT on four occasions *vs.* control group (waiting list)	Both CBT methods were superior to control group and induced adequate relief of global symptoms.
Ljótsson et al. (2010) Internet-delivered exposure and mindfulness based therapy for irritable bowel syndrome—a randomized controlled trial. *Behav Res Ther*. (35)	85 (85% female)	IBS symptom severity Quality of life Anxiety Depression General functioning	CBT *via* Internet *vs.* control group (online discussion forum)	CBT group: 42% decrease in IBS symptoms, control group: 12% increase in IBS symptoms.
Craske et al. (2011) A cognitive-behavioral treatment for irritable bowel syndrome using interoceptive exposure to visceral sensations. *Behav Res Ther*. (45)	110 (74% female)	Clinical symptoms	10 sessions of CBT or stress reduction training or attention control	CBT was superior to stress reduction training and attention control with regards to several domains; no difference between stress reduction training and attention control.
Bonnert et al. (2017) Internet-delivered cognitive behavior therapy for adolescents with irritable bowel syndrome: a randomized controlled trial. *Am J Gastroenterol*. (37)	101 (61% female)	Gastrointestinal symptoms Quality of life	10 weeks internet CBT *vs.* control group (wait list)	Greater improvement of gastrointestinal symptoms and quality of life in CBT compared to control group.
Lackner et al. (2018) Improvement in gastrointestinal symptoms after cognitive behavior therapy for refractory irritable bowel syndrome. *Gastroenterology*. (46)	436 (80% female)	Gastrointestinal symptoms	Standard CBT: 10 weeks: 1x 60 min/week or minimal therapist contact CBT: four sessions or education (four sessions)	Minimal contact CBT was more effective than education and as effective as standard CBT.
Everitt et al. (2019) Therapist telephone-delivered CBT and web-based CBT compared with treatment as usual in refractory irritable bowel syndrome: the ACTIB three-arm RCT. *Health Technol Assess*. (38)	558 (76% female)	IBS severity score Work and social adjustment scale	Telephone-delivered CBT: 9 weeks: 6x 1 h sessions + 2 x 1 h at months 4+8 *vs.* web-delivered CBT: 9 weeks: 3x 30 min telephone sessions + 2x 30 min at months 4+8 *vs.* treatment as usual	CBT increased capacity to cope with symptoms and negative emotions; both CBT arms induced improvement in IBS severity score at 3, 6, 12 months compared to TAU.
Zhao et al. (2019) Effect of cognitive behavior therapy combined with exercise intervention on the cognitive bias and coping styles of diarrhea-predominant irritable bowel syndrome patients. *World J Clin Cases*. (47)	57 (75% female)	Cognitive bias Coping styles	CBT + exercise *vs.* control group (drug therapy)	Greater improvement of cognitive bias and coping styles in CBT + exercise compared to control group.

CBT, cognitive behavioral therapy; GI, gastrointestinal; IBS, irritable bowel syndrome; n.s., not specified; TAU, treatment as usual; RCT, randomized controlled trial.

### Psychodynamic Psychotherapy

Psychodynamic psychotherapy focuses on intra- and interpersonal conflicts and how they contribute to the development and maintenance of symptoms. Psychodynamic psychotherapy leads to an improvement in IBS symptoms with a NNT of 4 (95% CI 2–20) (19). Therefore, also psychodynamic psychotherapy is recommended by the world gastroenterology organization (26).

A study from 1983 studied the impact of psychodynamic interventions on symptoms of IBS. A group received, additionally to a medical therapy, a psychodynamic intervention. Compared to the control group with medical therapy only, the intervention group showed a more pronounced improvement of symptoms after 3 months. The improvement was still observed after 1 year (49).

An RCT showed that a psychodynamic intervention is related to a reduction of interpersonal conflicts (**Table 4**). The reduction of interpersonal conflicts was a predictor for an improvement of health status in comparison to medical therapy (52). Nonetheless, also the control group with antidepressant medication showed a reduction of somatic symptoms.

**Table 4:** Randomized controlled studies investigating the effects of psychodynamic psychotherapy in patients with irritable bowel syndrome.

Study	Population	Variables	Intervention	Results
Svedlund et al. (1983) Controlled study of psychotherapy in irritable bowel syndrome. *Lancet*. (49)	101 (69% female)	Somatic symptoms	3 months: 10x 1 h session psychodynamic psychotherapy + medical treatment *vs.* control group (medical treatment)	Greater improvement of somatic symptoms in psychodynamic group; difference between both groups more pronounced after 1 year follow-up.
Guthrie et al. (1991) A controlled trial of psychological treatment for the irritable bowel syndrome. *Gastroenterology*. (50)	102 (74% female)	IBS symptoms	3 months: eight sessions psychodynamic therapy (plus relaxation plus medication) *vs.* control (medical treatment)	At 3 months greater improvement in diarrhea and abdominal pain in psychodynamic group compared to control.
Creed et al. (2003) The cost-effectiveness of psychotherapy and paroxetine for severe irritable bowel syndrome. *Gastroenterology (51)*	252 (80% female)	IBS symptoms Quality of life Health care costs	3 months: eight sessions of psychodynamic psychotherapy *vs.* paroxetine *vs.* control group (routine care)	Psychodynamic and paroxetine improved in global symptoms; during follow up psychotherapy was more cost efficient than paroxetine and control.
Hyphantis et al. (2009) Psychodynamic interpersonal therapy and improvement in interpersonal difficulties in people with severe irritable bowel syndrome. *Pain*. (52)	247 (80% female)	Interpersonal problems Abdominal pain Bowel symptoms Psychological distress Health status	Psychodynamic psychotherapy *vs.* antidepressant *vs.* control group (routine care)	Psychodynamic therapy induced a reduction of interpersonal conflicts; medical treatment improved somatic symptoms.

IBS, irritable bowel syndrome.

Another study compared the effects of a psychodynamic intervention with a treatment with paroxetin (selective serotonin reuptake inhibitor, SSRI). There was no significant difference related to pain reduction after 3 months. After 1 year both interventions improved the somatic component of quality of life. Overall, psychodynamic psychotherapy is a cost-effective alternative for a drug therapy of IBS (51).

### Hypnotherapy

Hypnotherapy is a method to focus on the perception of intestinal symptoms. The therapist is trying to impart bowel control to the patient and to achieve a change of the individual reaction on somatic symptoms (53). It was shown that the NNT with hypnotherapy is 5 (95% CI 3.5–10) (19). The world gastroenterology organization recommends hypnotherapy for patients with IBS refractory to drug treatment. However, although it shows more safety and tolerability compared to drug therapy, it may be labor-intensive and not always available (26).

A study from 1984 investigated the effect of hypnotherapy in patients with hard-to-treat IBS in comparison to supportive psychotherapy. The group with hypnotherapy showed a significant improvement of pain, flatulencies, changes in bowel habit, and general well-being. The follow-up after 3 months showed a persistence of the improvement (54). Another study showed that both hypnotherapy one-to-one sessions and group sessions induced a subjective relief associated with an improvement in quality of life, somatic and psychological symptoms (**Table 5**). The improvement continued for 12 months and there was no difference between the different types of sessions (61).

**Table 5:** Randomized controlled studies investigating the effects of hypnotherapy in patients with irritable bowel syndrome.

Study	Population	Variables	Intervention	Results
Whorwell et al. (1984) Controlled trial of hypnotherapy in the treatment of severe refractory irritable-bowel syndrome. *Lancet*. (55)	30 (87% female)	Gastrointestinal symptoms General well-being	Hypnotherapy *vs.* control (supportive psychotherapy)	Hypnotherapy group: significant improvement of gastrointestinal symptoms, no change in control group; benefit persisted after 3 months follow-up.
Galovski & Blanchard (1998) The treatment of irritable bowel syndrome with hypnotherapy. *Appl Psychophysiol Biofeedback*. (56)	12 (83% female)	IBS symptoms Anxiety	6 weeks: 1x 30 min to 1 h gut directed hypnotherapy/week *vs.* control group: symptom watching waiting list	Greater improvement of gastrointestinal symptoms in hypnotherapy group, decrease of anxiety.
Simrén et al. (2004) Treatment with hypnotherapy reduces the sensory and motor component of the gastrocolonic response in irritable bowel syndrome. *Psychosom Med*. (57)	26 (68% female)	IBS symptoms Barostat measurements	12 weeks: 1x 1 h session gut-directed hypnotherapy/week *vs.* control (supportive therapy)	More frequent improvement in global symptoms in hypnotherapy *vs.* control group; hypnotherapy reduced the sensory and motor component gastrocolonic response.
Lindfors et al. (2012) Effects of gut-directed hypnotherapy on IBS in different clinical settings—results from two randomized, controlled trials. *Am J Gastroenterol*. (58)	Study 1: 90 (79% female) Study 2: 48 (81% female)	IBS symptoms	Study 1: 12 weeks: 1x 1 h hypnotherapy/week + audiotapes for exercising at home *vs.* supportive therapy Study 2: hypnotherapy sessions in hospital *vs.* waiting list controls	Improvement of IBS symptoms in 3 months in both studies; greater improvement in hypnotherapy groups.
Moser G et al. (2013) Long-term success of gut-directed group hypnosis for patients with refractory irritable bowel syndrome: a randomized controlled trial. *Am J Gastroenterol*. (59)	90 (79% female)	Quality of life Psychological status IBS symptoms	12 weeks: 10x 45 min gut-directed hypnotherapy sessions + exercise at home + medical treatment *vs.* control group (medical treatment)	Gut-directed hypnotherapy was superior to medication therapy and showed a long-term effect.
Rutten et al. (2017) Home-based hypnotherapy self-exercises *vs.* individual hypnotherapy with a therapist for treatment of pediatric irritable bowel syndrome, functional abdominal pain, or functional abdominal pain syndrome: a randomized clinical trial. *JAMA Pediatr*. (60)	260 (72% female)	Pain frequency Intensity score	Cd group: 3 months hypnotherapy with 3 exercises/week *vs.* therapist group: 3 months hypnotherapy with six sessions	Cd hypnotherapy is not inferior to therapist hypnotherapy.
Flik et al. (2019) Efficacy of individual and group hypnotherapy in irritable bowel syndrome (IMAGINE): a multicentre randomised controlled trial. *Lancet Gastroenterol Hepatol*. (61)	354 (76% female)	Quality of life IBS symptoms	Individual hypnotherapy *vs.* group hypnotherapy *vs.* control group (education)	Improvement in life quality, somatic and psychological symptoms by hypnotherapy; no difference between individual or group therapies.

Cd, compact disc; IBS, irritable bowel syndrome.

In a study from 2003, 23 patients with IBS and rectal hyper-/hypo- or normal sensitivity were treated with hypnotherapy for 12 weeks and the sensory perception was compared with a healthy control group. The study showed that hypnotherapy can improve abnormal sensory perception in patients with IBS (62). Another study from 2004 investigated the gastrocolonic response of patients with IBS undergoing hypnotherapy and showed a reduction of the sensory and motor component of the gastrocolonic response (57). Hypnotherapy has also an effect on the processing and perception of visceral stimuli in patients with IBS. A study using fMRI suggested that hypnotherapy is able to normalize altered perception (63).

Lastly, a study showed that children with IBS benefit from a Cd-based therapy to the same extent as from therapeutic one-to-one sessions (60). Therefore, also hypnotherapy can be applied in a highly cost-efficient manner.

### Mindfulness-Based Therapy

Mindfulness-based therapy combines stress reduction and elements from CBT. The patients learn to perceive the complaints and to better cope with them (64). Due to the small number of studies investigating mindfulness-based therapies, no NNT has been calculated yet.

A prospective study investigated the effects from a stress reduction program on parameters like bowel complaints, quality of life, and gastrointestinal symptom-specific anxiety in patients with IBS. No effect was seen after 2 months, but after 6 months there was an improvement in quality of life and a reduction of symptom-specific anxiety. However, bowel-associated complaints did not change significantly (65). A randomized, controlled trial with 75 female IBS patients showed a reduction of gastrointestinal symptoms after 8 weeks of mindfulness-based therapy in comparison to the control group with social support only. The follow-up after 3 months showed that the reduction persisted associated with an improvement in quality of life and reduction of stress (64). Other studies with female IBS patients reported a significant improvement of symptom severity and quality of life after 8 weeks of mindfulness-based therapy compared to a control group. Mindfulness-based therapy reduced visceral sensitivity, mental stress, or over-evaluation of stress. Moreover, patients with IBS undergoing mindfulness training showed a nonreactivity to gut-focused anxiety and catastrophic appraisals compared to a social support control group (66).

Lastly, in a study population of 90% women and 10% men a mindfulness-based therapy was applied for 8 weeks (**Table 6**). The bowel complaints showed a significant reduction in comparison to control patients on the waiting list. The level of stress was reduced, but there was no significant difference in quality of life and mood between the two groups. The outcome after 6 months showed no difference between both groups due to a rebound of complaints (67). Therefore, mindfulness-based therapy might have to be supplemented by other treatments in order to exert a more sustained effect.

**Table 6:** Randomized controlled studies investigating the effects of mindfulness-based therapy in patients with irritable bowel syndrome.

Study	Population	Measured variable	Intervention	Results
Gaylord et al. (2011) Mindfulness training reduces the severity of irritable bowel syndrome in women: results of a randomized controlled trial. *Am J Gastroenterol*. (64)	75 (100% female)	Quality of life Visceral sensitivity index Treatment credibility scale	8 weeks: 1x 2 h mindfulness training/ week *vs.* support group	Greater reductions in IBS symptom severity after treatment and at 3 months follow up in mindfulness compared to support group.
Garland et al. (2012) Therapeutic mechanisms of a mindfulness-based treatment for IBS: effects on visceral sensitivity, catastrophizing, and affective processing of pain sensations. *J Behav Med*. (66)	75 (100% female)	IBS severity Quality of life	8 weeks: mindfulness training *vs.* social support	Mindfulness training promoted nonreactivity to IBS-associated anxiety and catastrophic appraisals.
Zernicke et al. (2013) Mindfulness-based stress reduction for the treatment of irritable bowel syndrome symptoms: a randomized wait-list controlled trial. *Int J Behav Med*. (67)	90 (90% female)	IBS symptom severity Quality of life Stress Mood	8 weeks: 1x 90 min/week mindfulness-based stress reduction *vs.* control group (waiting list)	Greater decrease in symptom severity in mindfulness group; benefit for overall symptoms persisted at 6 months follow-up.

IBS, irritable bowel syndrome.

### Relaxation Therapy

Relaxation therapies like progressive muscle relaxation or autogenic training aim to reduce perceived stress since stress can lead to a physiological arousal further increasing the somatic complaints and negatively influencing the communication between gut and brain (68). These therapies were shown to have a NNT of 6, however, with a broad range (95% CI 3–60) (19). This broad range likely also contributed to the assessment that relaxation therapy (alone) is not more effective than usual care in the relief of global IBS symptoms (26).

A randomized controlled trial showed the effect of a stress management program in comparison to peppermint oil. These patients learned different relaxation methods and 2/3 of them were able to reduce their pain attacks as well as their complaints. The reduction persisted up to 1 year follow-up (69). A comparison between medical therapy and progressive muscle relaxation showed that the relaxation method could reduce anxiety more effectively; however, there was no significant difference in reducing the somatic symptoms (70). A program of progressive muscle relaxation for 2 months at home led to an improvement of gastrointestinal symptoms compared to symptom control only. However, the study size was very small with 16 patients only (71).

A more recent trial studied the effects of relaxation methods (progressive muscle relaxation, breathing techniques) and training of emotional awareness and expression compared to waiting list patients. At the beginning the patients frequently showed a low emotional reaction to stress events and relationship conflicts. This can lead to avoidance behavior and chronic arousal. The emotional awareness and expression training reduced IBS symptom severity after 2 and 10 weeks compared to the control group. There was an improvement of quality of life in the emotional training group as well as in the group with relaxation methods. After 10 weeks the positive effects persisted; however, only in the relaxation group there was a significantly lower level of depressive and anxious symptoms (72).

A trial on young patients studied the effect of yoga exercises on IBS symptoms. The patients exercised yoga at home for 4 weeks. In comparison to patients on a waiting list, there was a reduced functional limitation, a lower avoidance behavior, and less anxiety symptoms (**Table 7**). However, there was no effect on depressive symptoms and on somatic symptoms (60). A recent trial compared the effect of yoga with the effect of a low-FODMAP diet. After 12 weeks yoga exercise at home and nutritional counselling in the control group, there was a significant reduction of the severity of gastrointestinal symptoms in both groups which persisted in the following year. It is to note that yoga also reduced anxiety symptoms (61).

**Table 7:** Randomized controlled studies investigating the effects of relaxation therapy in patients with irritable bowel syndrome.

Study	Population	Measured variable	Intervention	Results
Bennett & Wilkinson (1985) A comparison of psychological and medical treatment of the irritable bowel syndrome. *Br J Clin Psychol*. (70)	33 (70% female)	IBS symptoms Anxiety	Progressive muscle relaxation *vs.* medical treatment	Reduction of initial high anxiety levels in relaxation group only; IBS symptoms were reduced in both groups.
Lynch & Zamble (1989) A controlled behavioral treatment study of irritable bowel syndrome. *Behav Ther*. (73)	21 (67% female)	IBS symptoms Mood Self perception	8 weeks: 1x 2 h relaxation therapy /week and audio material for practicing twice at home *vs.* control group (waiting period)	Improvement of measured variables after treatment; benefit persisted for 5 months.
Shaw et al. (1991) Stress management for irritable bowel syndrome: a controlled trial. *Digestion*. (69)	35 (57% female)	IBS symptoms	6x 40 min sessions stress management program *vs.* control group (conventional therapy including antispasmodic)	2/3 of patients attending the stress program showed relief in symptoms and fewer attacks of less severity; benefit maintained for 12 months.
Blanchard et al. (1993) Relaxation training as a treatment for irritable bowel syndrome. *Biofeedback Self Regul*. (71)	23 (78% female)	Gastrointestinal symptoms	2 weeks: two sessions progressive muscle relaxation/week, 6 weeks: one session progressive muscle relaxation/week with regular home training *vs.* control group (monitoring)	Relaxation showed greater improvement in gastrointestinal symptoms than the symptom monitoring group.
Keefer & Blanchard (2001) The effects of relaxation response meditation on the symptoms of irritable bowel syndrome: results of a controlled treatment study. *Behav Res Ther*. (74)	13 (69% female)	IBS symptoms	6 weeks: 1x 30 min relaxation response meditation/week *vs.* control group (waiting list)	Meditation was superior to control.
Kuttner et al. (2006) A randomized trial of yoga for adolescents with irritable bowel syndrome. *Pain Res Manag*. (75)	28 (71% female)	Gastrointestinal symptoms Pain Functional disability Anxiety Depression	Yoga intervention: 1 h instruction, daily home practice over 4 weeks *vs.* control group (wait list)	Yoga group showed lower levels of functional disability, lower avoidance behavior and less anxiety symptoms compared to control.
van der Veek et al. (2007) Clinical trial: short- and long-term benefit of relaxation training for irritable bowel syndrome. *Aliment Pharmacol Ther*. (76)	98 (73% female)	IBS symptom severity Quality of life Frequency of doctor visits	4x 90 min sessions of relaxation therapy in small groups *vs.* control group (standard medical care)	Improvement in the measured variables by relaxation therapy compared to control; number needed to treat for long-term improvement was 5.
Shinozaki et al. (2010) Effect of autogenic training on general improvement in patients with irritable bowel syndrome: a randomized controlled trial. *Appl Psychophysiol Biofeedback*. (77)	21 (52% female)	IBS symptoms Anxiety Depression	8 weeks: 1x 30–40 min session autogenic training/week *vs.* control group (discussions)	Improvement of social functioning and bodily pain by autogenic training.
Boltin et al. (2015) Gut-directed guided affective imagery as an adjunct to dietary modification in irritable bowel syndrome. *J Health Psychol*. (78)	34 (76% female)	Symptom severity Quality of life	8 weeks: 1x 3 h session psychotherapy + guided affective imagery *vs.* control (no psychotherapy)	Reduction of symptom severity and improvement of quality of life by affective imagination.
Thakur et al. (2017) Emotional awareness and expression training improves irritable bowel syndrome: a randomized controlled trial. *Neurogastroenterol Motil*, (72)	106 (80% female)	Symptom severity Quality of life	2 weeks: 3x 50 min sessions relaxation therapy or emotional awareness/expression training or control (wait list)	Relaxation training reduced depressive symptoms; emotional awareness/expression training reduced IBS symptom severity and improved quality of life after 10 weeks follow-up while it did not reduce somatic symptoms.
Schumann et al. (2018) Randomised clinical trial: yoga *vs.* 6low-FODMAP diet in patients with irritable bowel syndrome. *Aliment Pharmacol Ther*. (79)	59 (n.s.)	Gastrointestinal symptoms Quality of life	12 weeks: two sessions/week yoga + exercise at home *vs.* control group (FODMAP)	Reduction of gastrointestinal symptoms in both groups; yoga reduced anxiety symptoms.

FODMAP, fermentable, oligo-, di-, monosaccharides and polyols; IBS, irritable bowel syndrome; n.s., not specified.

## Summary

IBS patients often report a great burden of disease associated with a significant impairment in quality of life. Psychotherapy is an important treatment column for patients with IBS with different procedures available, all of which are well to very well evidence-based by now. Consequently, the world gastroenterology organization states that CBT, hypnotherapy, and psychodynamic therapy are more effective in improving global symptoms than usual care (26).

It is to note that the current review also has limitations. First of all, only few studies were at low risk of bias which should be taken into account when interpreting the data. Moreover, some psychotherapeutic techniques were tested in few randomized studies so far; therefore, conclusions should be drawn with caution. Lastly, IBS is a heterogeneous disease which should be considered when performing a study and also when extrapolating the results to “real life” patients, especially those presenting to tertiary care centers which very often report (psychiatric) comorbidities.

Future perspectives of psychotherapy in IBS have also been investigated in few studies. A recent study showed that tele-hypnotherapy also leads to a reduction of pain, anxiety, and IBS severity in patients with IBS (80). Moreover, CBT offered *via* computer or telephone was superior to treatment as usual (38). Future studies should further explore these media as well as options for e-health interventions.

Taken together, it seems to be important to offer a multicomponent therapeutic strategy including psychoeducation, other psychotherapeutic interventions in addition to basal/drug therapy. These multicomponent approaches should be further investigated in controlled trials.

## Author Contributions

LH performed the literature search and wrote the first draft of the paper, AS planned and supervised the project as well as thoroughly revised the paper.

## Funding

This work was supported by funding of the German Research Foundation STE 1765/3-2 and Charité University Funding UFF 89/441-176 (AS).

## Conflict of Interest

AS is consultant for a&r Berlin, Boehringer-Ingelheim, Takeda, and Dr. Willmar Schwabe.

The remaining authors declares that the research was conducted in the absence of any commercial or financial relationships that could be construed as a potential conflict of interest.
